# CD151-enriched migrasomes mediate hepatocellular carcinoma invasion by conditioning cancer cells and promoting angiogenesis

**DOI:** 10.1186/s13046-024-03082-z

**Published:** 2024-06-06

**Authors:** Kangnan Zhang, Zhenhua Zhu, Rongrong Jia, NA Wang, Min Shi, Yugang Wang, Shihao Xiang, Qinghui Zhang, Ling Xu

**Affiliations:** 1 Department of Gastroenterology, Tongren Hospital, Shanghai Jiaotong University School of Medicine, Shanghai, 200336 China; 2 Key Laboratory for Translational Research and Innovative Therapeutics of Gastrointestinal Oncology, Tongren Hospital, Shanghai Jiao Tong University School of Medicine, Shanghai, 200336 China; 3 Key Laboratory of Cell Differentiation and Apoptosis of Chinese Ministry of Education, Department of Pathophysiology, Shanghai Jiao Tong University School of Medicine (SJTU-SM), Shanghai, 200001 China; 4 Department of Clinical laboratory, Tongren Hospital, Shanghai Jiao Tong University School of Medicine, Shanghai, 200336 China

**Keywords:** CD151, Migrasome, Liver cancer, Angiogenesis, Metastasis

## Abstract

**Background:**

The tetraspanin family plays a pivotal role in the genesis of migrasomes, and Tetraspanin CD151 is also implicated in neovascularization within tumorous contexts. Nevertheless, research pertaining to the involvement of CD151 in hepatocellular carcinoma (HCC) neovascularization and its association with migrasomes remains inadequate.

**Methods:**

To investigate the correlation between CD151 and migrasome marker TSPAN4 in liver cancer, we conducted database analysis using clinical data from HCC patients. Expression levels of CD151 were assessed in HCC tissues and correlated with patient survival outcomes. In vitro experiments were performed using HCC cell lines to evaluate the impact of CD151 expression on migrasome formation and cellular invasiveness. Cell lines with altered CD151 expression levels were utilized to study migrasome generation and in vitro invasion capabilities. Additionally, migrasome function was explored through cellular aggregation assays and phagocytosis studies. Subsequent VEGF level analysis and tissue chip experiments further confirmed the role of CD151 in mediating migrasome involvement in angiogenesis and cellular signal transduction.

**Results:**

Our study revealed a significant correlation between CD151 expression and migrasome marker TSPAN4 in liver cancer, based on database analysis of clinical samples. High expression levels of CD151 were closely associated with poor survival outcomes in HCC patients. Experimentally, decreased CD151 expression led to reduced migrasome generation and diminished in vitro invasion capabilities, resulting in attenuated in vivo metastatic potential. Migrasomes were demonstrated to facilitate cellular aggregation and phagocytosis, thereby promoting cellular invasiveness. Furthermore, VEGF-enriched migrasomes were implicated in signaling and angiogenesis, accelerating HCC progression.

**Conclusions:**

In summary, our findings support the notion that elevated CD151 expression promotes migrasome formation, and migrasomes play a pivotal role in the invasiveness and angiogenesis of liver cancer cells, thereby facilitating HCC progression. This finding implies that migrasomes generated by elevated CD151 expression may constitute a promising high-priority target for anti-angiogenic therapy in HCC, offering crucial insights for the in-depth exploration of migrasome function and a renewed comprehension of the mechanism underlying liver cancer metastasis.

**Supplementary Information:**

The online version contains supplementary material available at 10.1186/s13046-024-03082-z.

## Introduction

Primary liver cancer stands as the third leading cause of cancer-related mortality globally, with an annual incidence approaching one million cases [1]. While early-stage HCC can be addressed through interventions such as tumour resection, liver transplantation, and other surgical modalities, over 50% of HCC cases are diagnosed at advanced stages, with a 70% recurrence rate within the initial five years of treatment [2]. Current approaches to cancer treatment encompass surgical resection, liver transplantation, radiofrequency ablation, chemoembolization, targeted therapy, and immunotherapy [3–6]. However, HCC, characterised by heightened vascularity, presents challenges, with a majority diagnosed at intermediate to advanced stages exhibiting a high metastatic rate, resulting in an unfavorable prognosis [7]. Yet, the molecular mechanisms governing neovascularization in HCC remain incompletely elucidated.

Tetraspanins, widely expressed cell surface proteins [8], exert physiological roles in cellular functions and pathological conditions [9, 10], including metastasis and pathological angiogenesis [11]. Migrasome, a recently identified organelle primarily comprised of the tetraspanin 4 (TSPAN4) protein family, has emerged as a significant entity. TSPAN4 serves as a specific marker for migrasome, with its expression level influencing migrasome formation [12]. However, the precise functions of migrasome are not yet fully comprehended. Existing studies indicate their roles in embryonic development [13] and mitochondrial quality control [14, 15], with implications in cholesterol metabolism through mitochondrial proliferation [16, 17]. Furthermore, migrasomes are implicated in information transmission and intercellular signal integration [18]. Observations using two-photon synthetic aperture microscopy have demonstrated migrasomes’ capability in facilitating direct intercellular communication [19]. In cases of traumatic brain injury (TBI), it has been observed that neutrophils within the peripheral immune system can convey signals to microglia via migrasomes [19]. Recent investigations have highlighted the influence of migrasomes in creating an environment conducive to angiogenesis and enhancing vascular formation. Studies have shown that migrasomes enriched with angiogenic factors such as VEGFA and CXCL12 contribute to the formation of capillary membranes in chick embryos and the recruitment of monocytes [18]. Additionally, migrasomes serve as chemotactic cues, intensifying the recruitment of monocytes through a feed-forward loop to promote angiogenesis [20]. Given the essential role of angiogenesis in tumor metastasis, these findings suggest migrasomes might play a significant part in tumor progression. Nevertheless, related research is currently lacking.

CD151, another member of the tetraspanin family, is linked to highly invasive cancer cells, and its overexpression correlates with poor prognoses in various cancers, including lung cancer [21], colorectal cancer [22], prostate cancer [23], and liver cancer [8]. CD151 influences crucial cellular functions such as cell communication, wound healing, platelet aggregation, cell movement, and angiogenesis by binding with integrin and non-integrin proteins. Elevated CD151 expression is associated with increased VCAM-1 expression, ultimately fostering cell adhesion [24]. Additionally, CD151 participates in the collective cell migration process, promoting the polarization of epithelial cells and reorganization of the actin cytoskeleton by interacting with integrins and junctional adhesion molecule (JAM)-A, facilitating the recruitment of signaling molecules [25]. Unquestionably, CD151 plays a pivotal role in angiogenesis and cancer metastasis.

Our study delves into altering CD151 expression levels to investigate the secretion of migrasome and the invasive properties of liver cancer. We explore the role of migrasome in HCC-related neovascularization and metastasis, ultimately revealing that elevated CD151 expression upregulates migrasome expression in liver cancer. The generated migrasome acts as a signaling locator, enhancing the invasive properties of liver cancer cells. Moreover, the Vascular Endothelial Growth Factor (VEGF)-rich migrasome promotes angiogenesis, rendering liver cancer more susceptible to metastasis. This study unveils the significant influence of CD151 through migrasome on the vascular metastasis of cancer cells in liver cancer, providing crucial insights for further understanding the functions of migrasome and liver cancer through vascular metastasis.

## Methods

### Data collection and processing

The acquisition and processing of RNA-seq data and clinical data from patients with Liver Hepatocellular Carcinoma (LIHC) were conducted using resources such as The Cancer Genome Atlas (TCGA, https://portal.gdc.cancer.gov), Gene Expression Omnibus (GEO, https://www.ncbi.nlm.nih.gov/gds), and International Cancer Genome Consortium (ICGC, https://dcc.icgc.org/). The expression data for liver cancer cell lines were sourced from the DepMap portal (DepMap, https://depmap.org/portal/). Differential expression analysis was performed on LIHC samples, distinguishing between tumour and normal samples. Among the 33 types of tumours, only 17 types contained more than five pairs of tumour and normal samples. Consequently, differential expression analysis was restricted to these 17 types, with the ratio of the average expression value of tumour samples to that of normal samples representing the fold change. The t-test was employed to derive the associated p-value.

Upon integrating the expression data of tetraspanin-related genes with clinical information, risk ratios relative to Overall Survival (OS) were calculated for LIHC using Cox regression analyses to categorise samples into high or low risk. LIHC samples were dichotomised into two groups based on the optimal cutoff value, and a log-rank test was utilised to determine the associated p-value. Kaplan-Meier survival curves for OS were constructed for tetraspanin-related genes significant to LIHC, and subjected to the log-rank test. The graphical representation was achieved using the R package “survival.”

### Cell culture and transfection

HCCLM3 and MHCC97H cells were established at the Liver Cancer Institute (Zhongshan Hospital, Fudan University, Shanghai, China), and Hep3B, Huh7, HepG2 cells were procured from the American Type Culture Collection (ATCC). These cell lines, with STR identification reports, were consistently maintained.

To establish the HCCLM3-TSPAN4-GFP stable cell line, HCCLM3 cells underwent transfection with lentivirus, followed by selection with Blasticidin. The HCCLM3-CD151-RFP, HCCLM3-TSPAN4-RFP, Hep3B-TSPAN4-RFP stable cell lines were generated by transfecting HCCLM3 cells with CD151-RFP/TSPAN4-RFP vector, and Hep3B cells with TSPAN4-RFP vector, followed by selection with puromycin.

### Animal experiments

Six-week-old male athymic BALB/C nu/nu mice were randomly allocated to groups prior to inoculation. Mice were housed in a standard animal laboratory with unrestricted access to food and water, under constant environmental conditions with a 12-hour dark-light cycle. CD151 shRNA or Mock shRNA infected HCCLM3-luc/MHCC97H-luc cells (1 × 10^7) suspended in 0.2 mL serum-free culture medium were injected into the upper flank region of nude mice. Sacrifice occurred four weeks later, and tumors were harvested and measured for volume. Tumor volume was calculated using the formula: volume (mm^3) = (width)^2 × length/2.

An orthotopic model was constructed by producing tumors as described above. After two weeks, tumors were sectioned into small pieces of approximately 1.0 mm^3 and orthotopically transplanted into the livers of nude mice. These mice were allowed to grow for three months, and D-Luciferin was intraperitoneally injected. The emitted photon signal, detectable using Bruker MS FX PRO In vivo Imager, facilitated quantification of the total tumor burden. This technology enabled the assessment of tumor development ex vivo. The colour gradient represented the in vivo size of the tumor. Mice were then euthanised by cervical dislocation, and the livers were resected and photographed using a high-definition digital camera. All procedures complied with the Animal Committee of Tongren Hospital guidelines (approval number: A2023-119-01). The animal experiments in this study were strictly conducted in accordance with the guidelines provided by the Ministry of Science and Technology of the People’s Republic of China regarding the humane treatment of experimental animals, among other regulations.

### Preparation of small interfering RNA (siRNA)

CD151 (Human) siRNA were synthesized by KeyGEN BioTECH (China). Sequences of the three synthesized oligonucleotides are: CD151si1 sense: 5′-GCUGGAGAUCAUCGCUGGUAUTT-3′, CD151 si1 antisense: 5′-AUACCAGCGAUGAUCUCCAGCTT-3′; CD151si2 sense: 5′- CCCUCAAGAGUGACUACAUCATT-3′, CD151 si2 antisense: 5′- UGAUGUAGUCACUCUUGAGGGTT-3′; CD151si3 sense: 5′- GCCUCAAGUACCUGCUGUUUATT-3′, CD151 si3 antisense: 5′- UAAACAGCAGGUACUUGAGGCTT-3′.

### RNA interference, RNA isolation, and real-time PCR

Plasmids expressing short hairpin RNA (sh-RNA) targeted the following sequences: CD151-shRNA-1: 5′-GCCTCAAGTACCTGCTGTTTA-3′; CD151-shRNA-2: 5′-ACCTGCTGTTTACCTACAATT-3′; CD151-shRNA-3: 5′-CCCTCAAGAGTGACTACATCA-3′. A non-target shRNA (Scramble shRNA) against the human genome served as the control. Total RNA was extracted from cells using Trizol reagents (Invitrogen, Shanghai). The isolated mRNAs were reverse transcribed into complementary DNA (cDNA) using the Promega Reverse Transcription System (Madison, WI, USA) with Oligo dT priming. Real-time PCR was conducted using SYBR Green Premix Ex Taq (Takara, Japan) on a Light Cycler 480 (Roche, Switzerland). GAPDH mRNA levels were utilised as an internal control. Gene expression differences were determined using the 2-ΔΔCt method and expressed as fold-changes. PCR conditions comprised an initial holding period at 95 °C for 5 min, followed by a two-step PCR program of 95 °C for 5 s and 60 °C for 30 s for 50 cycles. Q-PCR primer sequences for CD151 were as follows: forward, 5′- AGACCATGCCTCCAACATCTA-3′, and reverse, 5′- CACAGGCAATGCCGATCC-3′. GAPDH primer sequences for Q-PCR were as follows: forward, 5′- AGGTCGGAGTCAACGGATTTG′, and reverse, 5′- CATCGCCCCACTTGATTTTG-3′.

### Western blot

Total proteins were extracted using the Whole Protein Extraction Kit (KGP200, KeyGen BioTECH, China), and protein concentrations were determined using a BCA protein assay kit (KGPBCA, KeyGen BioTECH, China). Separated samples were subjected to 10% SDS-PAGE and subsequently transferred to PVDF membranes. The following antibodies were used: CD151 (1:1000, sc-271,216, Santa Cruz Biotechnology, USA), β-actin (1:1000, sc-47,778, Santa Cruz Biotechnology, USA), EOGT (1:1000, ab190693, Abcam, USA), TSPAN4 (1:1000, ab181995, Abcam, USA), TSPAN7 (1:1000, 18695-1-AP, Proteintech, USA), Integrin α5 (1:1000, 4705, Cell Signaling Technology, USA), PIGK (1:1000, ab201693, Abcam, USA), PGCP (CPQ) (1:1000, ab96159, Abcam, USA), CD9 (1:1000, sc-13,118, Santa Cruz Biotechnology, USA), CD63 (1:1000, sc-5275, Santa Cruz Biotechnology, USA).

### Confocal imaging

Cells were cultured in 35 mm confocal dishes for 10–12 h, fixed with 4% paraformaldehyde, and stained with 1 µg/ml WGA488 (W11261, Life Technologies, USA) for 15 min (tissue sections were stained with 2 µg/ml for 30 min). Confocal images were acquired using a NIKON A1RSiHD25 laser scanning confocal microscope at 1024 × 1024 pixels. For live-cell imaging, cells were cultured in fibronectin-precoated confocal dishes for 4–6 h before imaging. The cells were maintained at 37 °C with 5% CO2 and monitored using a NIKON A1 microscope at 1024 × 1024 pixels.

### Migrasome purification

Migrasome purification was conducted through iodixanol sucrose density-gradient centrifugation using an Optiprep kit (LYSISO1, Sigma-Aldrich). The samples underwent sequential centrifugation steps: 1,000 g for 5 min at 4 °C to eliminate cell bodies, 4,000 g for 20 min at 4 °C to remove cell fragments, and finally 20,000 g for 20 min at 4 °C. The pellet containing the crude migrasome fraction was re-suspended, lysed in extraction buffer (Sigma-Aldrich), and fractionated at 150,000 g for 4 h at 4 °C in a multi-step Optiprep dilution gradient. The gradient included concentrations of 3%, 5%, 8%, 12%, 16%, 19% (sample), 22.5%, and 27%. Fractions were collected and added to 500 µl PBS, followed by centrifugation at 20,000 g for 30 min at 4 °C. The pellet was washed once with PBS, centrifuged at 4 °C, 2,000 g for 10 min, and the supernatant was collected. Further centrifugation at 4 °C, 20,000 g for 30 min resulted in migrasomes for subsequent experiments.

### Transmission Electron Microscopy

Cells were cultured in 35 mm dishes precoated with fibronectin (10 mg/ml). After 10–12 h, cells were pre-fixed using a 1:1 ratio of growth medium to 2.5% glutaraldehyde for 5 min at room temperature. Subsequently, cells were fixed with 2.5% glutaraldehyde in PB buffer for 2 h at room temperature, washed three times with PBS, and dehydrated in an ascending gradual series of ethanol (50%, 70%, 90%, 95%, and 100%) for 8 min each. Samples were infiltrated and embedded in SPON12 resin. After polymerisation for 48 h at 60 °C, ultrathin Sect. (70 nm thick) were cut using a diamond knife, picked up with Formvar-coated copper grids (100 mesh), and double-stained with uranyl acetate and lead citrate. Subsequently, air-dried samples were examined using a transmission electron microscope H-7650B at an acceleration voltage of 80 kV.

### Transwell invasion assay

Transwell inserts with 8-µm pore size membranes, coated with Matrigel (Corning, 354,277, USA), were allowed to solidify. A total of 1.5 × 10^5 cells, suspended in serum-free medium, were seeded into the upper chambers, while the lower chambers contained medium with 10% FBS as a chemoattractant. After 48 h of incubation, non-invasive cells on the upper membrane surface were removed, and invasive cells on the lower surface were fixed with 4% paraformaldehyde and stained with crystal violet. Images were captured, and migrated cells were quantified using an inverted microscope.

### Scratch assay

A scratch assay was utilised to assess cell migration. Cells were seeded in 6-well plates and allowed to reach confluence. A sterile 200-µL pipette tip was used to create a straight scratch across the cell monolayer. After washing with phosphate-buffered saline (PBS) to remove detached cells and debris, images of the scratch were captured immediately after scratching (0 h) and at designated time points during the cell migration process using an inverted microscope. ImageJ software facilitated measuring the scratch width, allowing quantification of cell migration.

### Chemotaxis experiments

Chemotaxis experiments were conducted using Transwell inserts with 8-µm pore size membranes. A cell suspension of 1.0 × 10^5 cells in serum-free medium was seeded into the upper chambers, while the lower chamber was filled with medium containing 10% FBS and migrasomes extracted from highly invasive liver cancer cells as a chemoattractant. After a 24-hour incubation, cells on the upper membrane surface were removed with a cotton swab. The invasive cells on the lower surface were fixed with 4% paraformaldehyde and stained with crystal violet. Images were captured, and migrated cells were quantified using an inverted microscope.

### Transmission electron microscopy

For transmission electron microscopy, samples were initially pre-fixed using a 1:1 ratio of growth medium to 2.5% glutaraldehyde at 4 °C overnight. Subsequently, samples were fixed with 2.5% glutaraldehyde in PB buffer for 2 h at room temperature, washed three times with PBS, and dehydrated in an ascending gradual series of ethanol (50%, 70%, 90%, 95%, and 100%) for 8 min each. Following infiltration with and embedding in SPON12 resin, 70-nm-thick ultrathin sections were cut using a diamond knife and picked up with Formvar-coated copper grids (100 mesh). Double staining with uranyl acetate and lead citrate was performed, and after air drying, samples were examined with a transmission electron microscope H-7650B at an acceleration voltage of 80 kV.

### Field emission scanning electron microscopy

For field emission scanning electron microscopy, samples were fixed with 2.5% glutaraldehyde in PB buffer overnight at 4 °C, washed three times with PB buffer, and post-fixed with 1% osmium containing 1.5% potassium ferrocyanide for 60 min at room temperature. Subsequently, all samples were dehydrated with a graded series of ethanol (50%, 70%, 90%, 95%, and 100%) for 8 min each. After changing ethanol with tert-Butanol, samples were frozen at − 20 °C, then dried with a freeze-drier. The dried samples were coated with an approximately 10-nm-thick gold film by sputter coating before examination with a field emission scanning electron microscope using an SE detector at an acceleration voltage of 3 kV.

### ELISA

The concentration of VEGF protein in HCC cell culture medium was determined by ELISA using the Human VEGF Quantikine ELISA Kit (DVE00, R&D Systems) following the manufacturer’s instructions. Absorbance of the samples was determined using the Synergy H4 Hybrid Reader (Biotek, Winooski, VT).

### Angiogenesis

Human umbilical vein endothelial cells (HUVECs) were cultured for 24 h and used for the angiogenesis assay. Cells were synchronized by incubating them in ECM containing 0.1% FBS for 12 h. 96-well plates were coated with Matrigel Basement Membrane Matrix (BD Biosciences; diluted in basal ECM at a ratio of 1:1; 20 µl mixture per well) and incubated at 37 °C for 30 min to allow gelation. HUVECs were plated at a density of 5 × 10^4 cells per well. After 4 h of incubation at 37 °C with 5% CO2, pictures were captured with a light microscope.

### Tissue microarray and immunohistochemistry

Tissue microarrays were meticulously crafted by Shanghai Zhuoli Biotechnology Co., Ltd (NO. ZL-LVC1801, Zhuoli Biotechnology Co, Shanghai, China), featuring two microarray chips (TMA) housing 90 pairs of tumours and their corresponding adjacent tissues. Acquisition of these samples was carried out with due approval from the hospital. The tissue samples underwent an initial deparaffinization process lasting 1 h, followed by dehydration in ethanol. To quench endogenous peroxidase, a 3% H2O2 treatment was administered. Subsequently, the arrays were subjected to a 20–30 min blocking step using 10% normal goat serum and incubated overnight at 4 °C with primary antibodies (CD151, sc-271,216, Santa Cruz Biotechnology; CD31, 66065-2-Ig, Proteintech). Post PBS washes, the tissue samples were exposed to HRP-conjugated secondary antibody for 1 h at room temperature. Positive signals were visualised using the DAB kit, which reacts with the HRP substrate. Following PBS washes and ethanol dehydration, the tissue slides were meticulously sealed and mounted for microscopic examination.

### Statistical analyses

All experiments underwent meticulous repetition three times, and the data is eloquently presented as mean ± standard deviation (SD). Statistical analysis was executed with GraphPad Prism 6.0 software. Group differences were calculated using Student’s t-test or one-way ANOVA with a Tukey post-hoc test. A significance level of *P* < 0.05 was adopted, and distinctions were considered statistically significant (* *P* < 0.05, ** *P* < 0.01, and *** *P* < 0.001).

## Results

### CD151 influences the formation of migrasomes and can serve as a marker for migrasomes in HCC

**Fig. 1:**
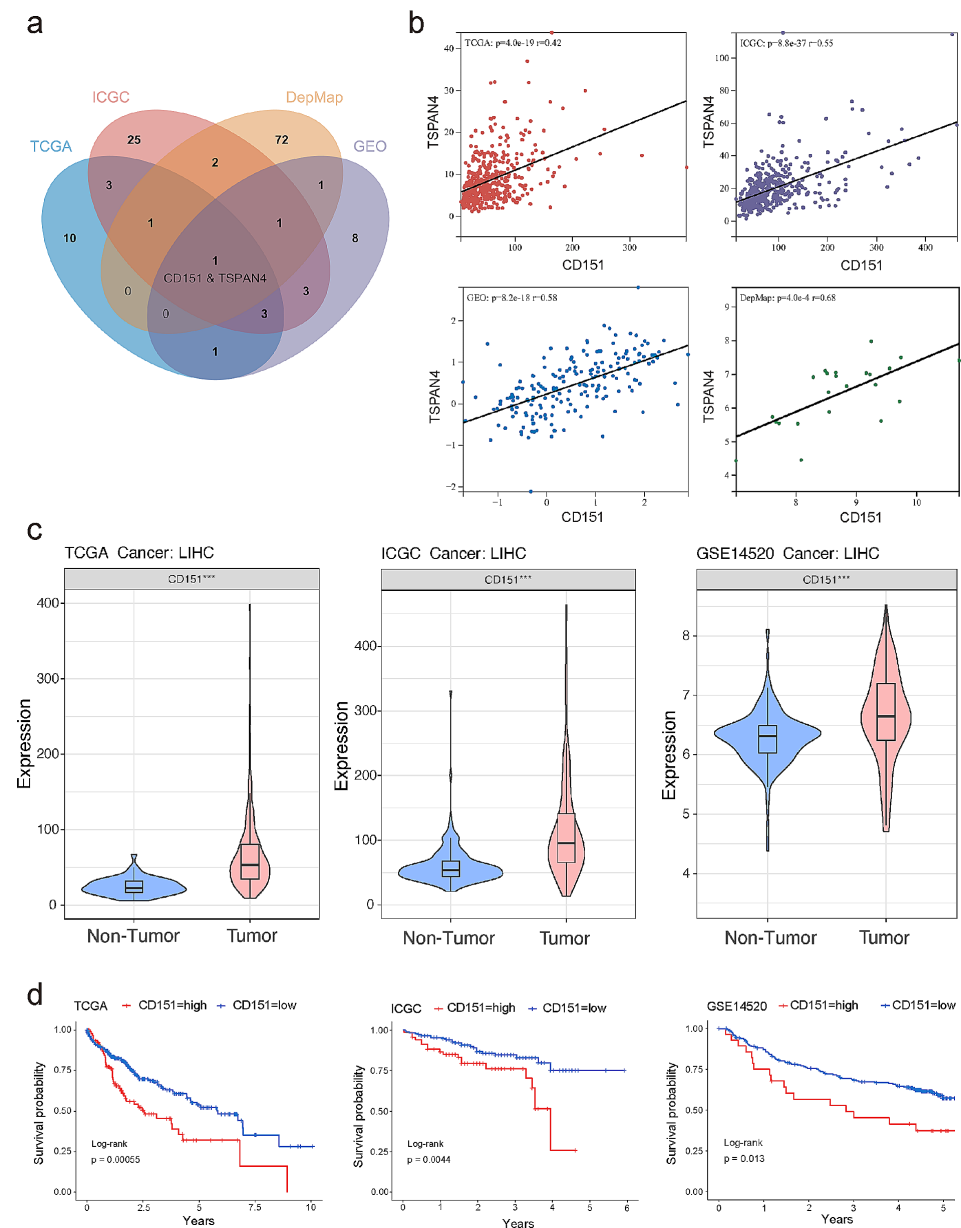
Correlation analysis of the tetraspanin family in liver cancer. **a**: Correlation analysis of the tetraspanin family in TCGA-LIHC, ICGC-LIRI, GSE1898, and DepMap-HCC cell lines datasets, depicting genes with correlation |r| > 0.4 in a Venn diagram. **b**: The heatmap presents the correlation of mRNA expression between CD151 and TSPAN4 in TCGA-LIHC, ICGC-LIRI, GSE1898, and DepMap-HCC cell lines datasets via the Corrplot package. **c**: Differential expression analysis of CD151 in non-tumor l and tumor samples from TCGA-LIHC, ICGC-LIRI, and GSE14520 datasets. **d**: Kaplan Meier curves depict the survival differences between normal and tumor samples for the CD151 gene in TCGA-LIHC, ICGC-LIRI, and GSE14520 datasets. The blue line represents non-tumor, while the red line represents tumor. *p < 0.05; **p < 0.01; ***p < 0.001; ns, not significant

Tetraspanins (TM4SF) exhibit the ability to bind to themselves and/or cell surface molecules, forming Tetraspanin Enriched Microdomains (TEMs). This network, involving four-transmembrane proteins, regulates essential cellular processes, including signal transduction, adhesion, migration, proliferation, and differentiation [26]. Extensive studies highlight the impact of Tetraspanins on tumour cell behaviour, influencing migration, invasion, angiogenesis, and epithelial-mesenchymal transition (EMT), thus contributing to tumour development [27, 28]. Given their potential role, we explored the mRNA expression data of the tetraspanin family genes in various datasets, including LIHC (TCGA), GSE1898 (GEO), LIRI (ICGC), and DepMap (HCC cell lines). A correlation analysis revealed a notable association between “CD151 & TSPAN4” across all datasets (Correlation coefficient | r |>0.4), indicating their heightened correlation in liver cancer (Fig. 1a, b; Table S1-4). Previous research underscores CD151’s role in enhancing migration and invasion in liver cancer cells [29]. Differential expression and survival analyses of CD151 in TCGA-LIHC, ICGC-LIRI, and GSE14520 affirmed its elevated expression in tumour samples with a corresponding correlation to poor prognosis (Fig. 1c, d). TSPAN4, identified as a marker for migrasomes [30], emerged as a potential player in liver cancer metastasis due to its significant correlation with CD151.

**Fig. 2:**
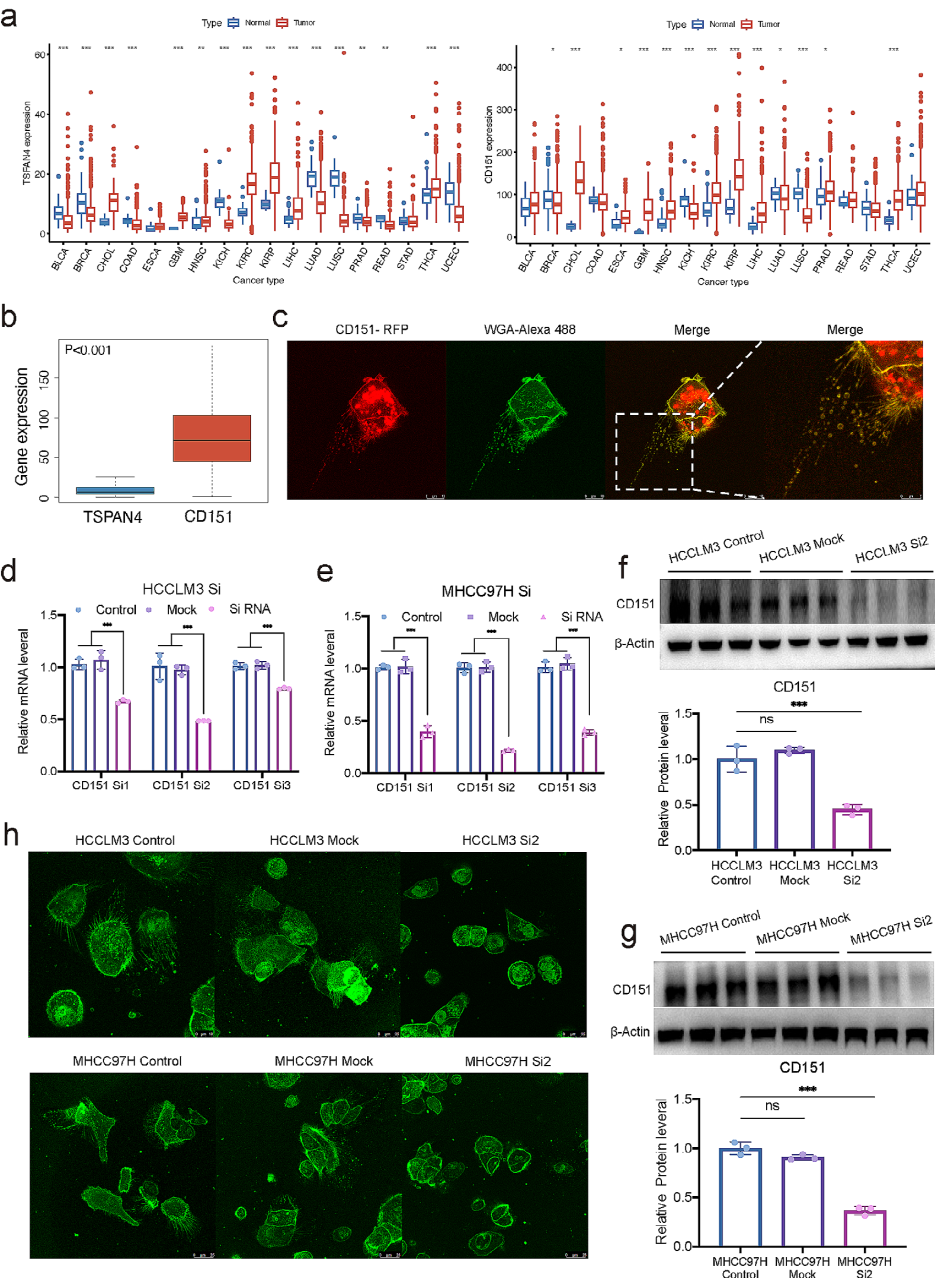
The expression of CD151 influences migrasome generation. **a**: mRNA expression levels of CD151 and TSPAN4 in tumor and non-tumor tissues across different tissues from the TCGA database. **b**: mRNA expression levels of CD151 and TSPAN4 in tumor tissues from the TCGA database. **c**: Confocal microscopy observation of HCCLM3 cells stably expressing CD151-RFP cultured for 12 h. **d**: RT-qPCR analysis of CD151 mRNA expression levels in HCCLM3 cells transfected with CD151 siRNA or Mock and untreated HCCLM3-Control cells. **e**: RT-qPCR analysis of CD151 mRNA expression levels in MHCC97H cells transfected with CD151 siRNA or Mock and untreated MHCC97H-Control cells. **f**: Western blot analysis of the impact of CD151 siRNA on CD151 protein levels in HCCLM3 cells. **g**: Western blot analysis of the impact of CD151 siRNA on CD151 protein levels in MHCC97H cells. **h**: Liver cancer cells cultured for 12 h, stained with 1 µg/ml WGA-Alexa 488, and observed under confocal microscopy. *p < 0.05; **p < 0.01; ***p < 0.001; ns, not significant

While migrasomes have been recognized as signalling organelles generated during cell movement, their expression and significance in various cancers remain poorly understood. Our investigation into TSPAN4 and CD151’s mRNA expression across different cancers highlighted their elevated levels in tumours (Fig. 2a), with CD151 consistently surpassing TSPAN4 across various tissues (Fig. 2b). To discern the relationship between CD151 and migrasomes, we constructed CD151-SiRNA and conducted RNAi KD experiments on liver cancer cells (HCCLM3 and MHCC97H). The effective knockdown of CD151 was confirmed through qPCR and Western blot (Fig. 2d-g). Subsequent staining with WGA-Alexa 488 revealed a significant reduction in migrasome formation in CD151 knockdown cells (Fig. 2h). Moreover, stable expression of CD151-RFP in HCCLM3 cells, along with WGA-Alexa 488 staining, substantiated the co-localization of CD151 within migrasomes (Fig. 2c). These experiments collectively suggest that CD151 expression in liver cancer cells plays a crucial role in migrasome generation, with CD151 acting as a label within these organelles.

### CD151 expression may affect the invasiveness of liver cancer cells through migrasome

**Fig. 3:**
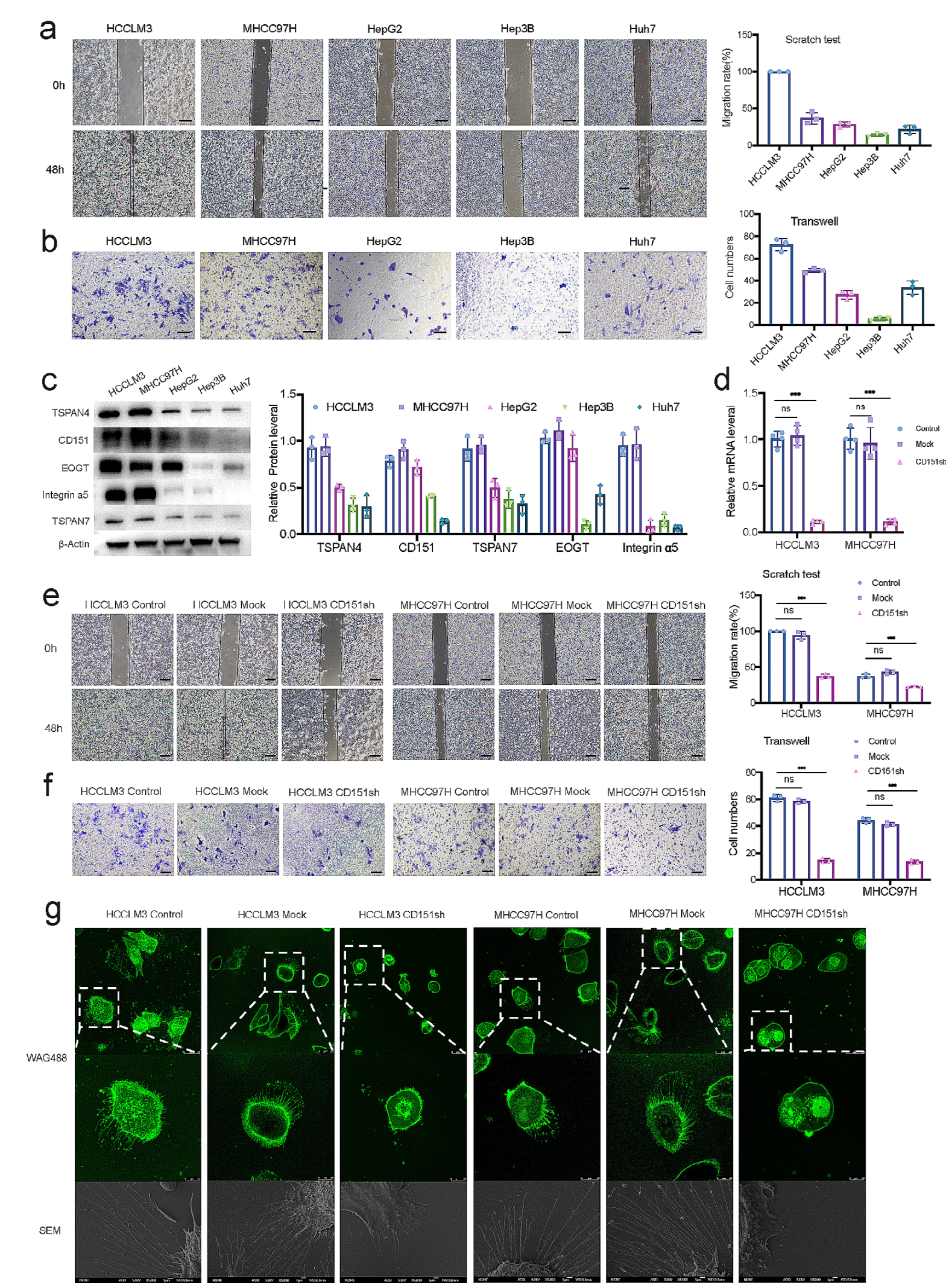
Association of CD151 with the migration and invasion capabilities of liver cancer cells. **a**: Scratch healing abilities of different liver cancer cell lines. **b**: Exploration of the invasion capabilities of different liver cancer cell lines through Transwell experiments. **c**: Expression levels of migrasome markers in different liver cancer cell lines studied through Western blot. **d**: RT-qPCR analysis of CD151 mRNA expression levels in HCCLM3/MHCC97H cells transfected with CD151 shRNA or Mock and untreated HCCLM3/MHCC97H (Control) cells. **e**: Impact of CD151 shRNA on the scratch healing abilities of HCCLM3/MHCC97H cell lines. **f**: Exploration of the impact of CD151 shRNA on the invasion capabilities of HCCLM3/MHCC97H cell lines through Transwell experiments. **g**: Observation of HCC cells affected by CD151 shRNA using SEM. **h**: HCC cells affected by CD151 shRNA cultured for 12 h, stained with 1 µg/ml WGA-Alexa 488, and finally observed under confocal microscopy. *p < 0.05; **p < 0.01; ***p < 0.001; ns, not significant

Previous investigations have affirmed the influence of CD151 on migrasome generation; however, the precise ramifications of CD151 on migrasome dynamics and its implications for the invasiveness of liver cancer cells remain elusive. Employing five distinct cell lines (HCCLM3, MHCC97H, HepG2, Hep3B, and Huh7), we assessed their migratory capabilities through scratch assays (Although HepG2 is a hepatoblastoma cell line, we included it in the experiments to compare the invasiveness and metastatic potential of liver-related tumor cell lines). Results revealed HCCLM3 and MHCC97H as the most proficient migrators, displaying migration rates of 100%±0 and 37.1%±2.5%, respectively (Fig. 3a). Furthermore, in vitro invasion assays highlighted HCCLM3 and MHCC97H as possessing the highest invasion capacities, with penetrated cell numbers of 72 ± 5 and 49 ± 2, respectively (Fig. 3b). Subsequent evaluation of CD151 expression in these cell lines unveiled heightened levels in HCCLM3 and MHCC97H, correlating with their enhanced migration and invasion abilities. Intriguingly, the expression of migrasome markers [31] (TSPAN4, TSPAN7, EOGT, and Integrin α5) also exhibited an elevation in these two cell lines (Fig. 3c). This observation led us to hypothesize that liver cancer cells with heightened invasiveness may express a greater abundance of migrasomes.

To validate this hypothesis, stable cell lines with reduced CD151 expression (HCCLM3-CD151sh and MHCC97H-CD151sh) were generated. The stable downregulation of CD151 was confirmed through quantitative real-time polymerase chain reaction (qRT-PCR) (Fig. 3d). Subsequent scratch assays demonstrated a significant decrease in migration rates for HCCLM3-CD151sh and MHCC97H-CD151sh cells (29.1%±1.3% and 22.6%±0.6%, respectively) compared to their respective controls (Fig. 3e). Additionally, in vitro invasion assays underscored a notable reduction in the invasive capacities of cells with low CD151 expression (Fig. 3f). The confocal microscopy, utilizing 1 µg/ml WGA-Alexa 488 staining, and scanning electron microscopy (SEM) further corroborated a substantial decline in migrasome generation in HCCLM3-CD151sh and MHCC97H-CD151sh cells, aligning with the overall decrease in invasiveness (Fig. 3g). Consequently, these findings reinforce the assertion that CD151 expression significantly influences both migrasome generation and the invasiveness of liver cancer cell lines.

### The migrasome acts as a localization signal, influencing and conditioning the invasion of liver cancer cells

**Fig. 4:**
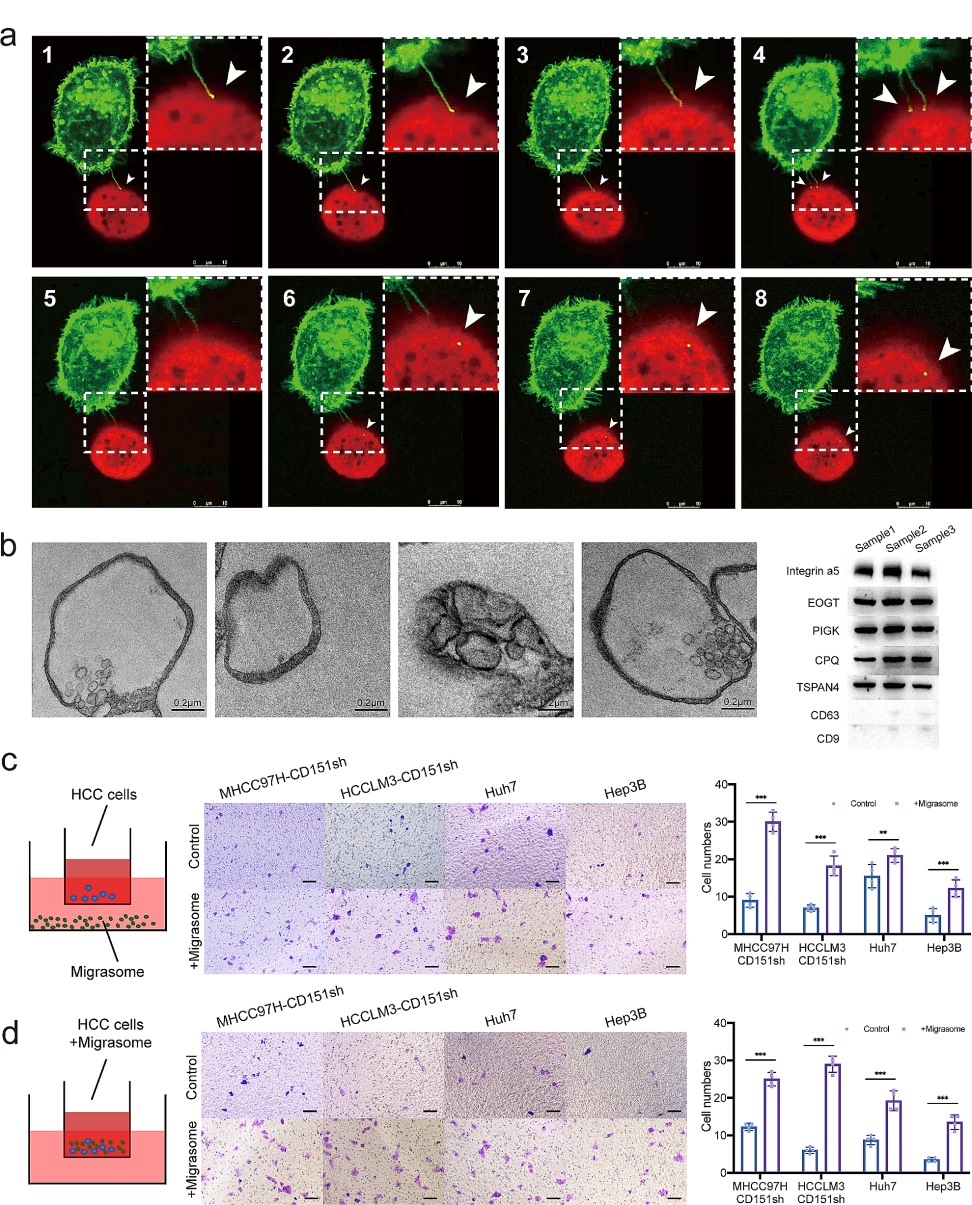
Modes of transmission and action of migrasomes. **a**: HCCLM3-TSPAN4-GFP cells and Hep3B-TSPAN4-RFP cells were co-cultured, and images were captured under confocal microscopy to observe the transfer of migrasomes between cells. Green: HCCLM3-TSPAN4-GFP cells; Red: Hep3B-TSPAN4-RFP. **b**: Observation of enriched migrasomes using scanning electron microscopy (SEM). Additionally, Western blot (WB) experiments validate markers in the extracted migrasomes. **c**: Chemoattractant effect of migrasomes from highly invasive liver cancer cells on less invasive liver cancer cells. **d**: Taming effect of migrasomes from highly invasive liver cancer cells on less invasive liver cancer cells, enhancing invasiveness. *p < 0.05; **p < 0.01; ***p < 0.001; ns, not significant

Prior investigations have elucidated the involvement of migrasomes in substance delivery [32] and intercellular signal integration [18, 19]. In this study, we establish a correlation between the production of migrasomes by liver cancer cells and their invasive potential. However, the mechanism through which migrasomes facilitate this invasiveness, specifically through intercellular transfer, remains unexplored. To delve into the intricacies of migrasome-mediated substance delivery between cells, we co-cultured HCCLM3-TSPAN4-GFP cells and Hep3B-TSPAN4-RFP cells, continually capturing images under confocal microscopy. Our observations unveiled that migrasomes between tumour cells can traverse into adjacent cells through engulfment, as demonstrated in Video S1. Migrasomes generated by HCCLM3-TSPAN4-GFP cells were observed to be engulfed by neighbouring Hep3B-TSPAN4-RFP cells (Fig. 4a).

To better understand how cells locate migrasomes, we isolated these vesicles from highly invasive HCCLM3 cells and confirmed their presence through Transmission Electron Microscopy (TEM) and Western blot (WB) analyses, verifying migrasome-specific markers [31]. Additionally, we utilized CD9 and CD63 [33, 34]Western blot results to demonstrate that the vesicles isolated were not exosomes (Fig. 4b). Introducing migrasomes under the Transwell chamber, and incorporating less invasive liver cancer cells like MHCC97H-CD151sh, HCCLM3-CD151sh, Huh7, and Hep3B into the chamber, revealed a marked increase in the number of cells traversing the chamber towards the cells below upon the addition of migrasomes (Fig. 4c). This observation suggests that migrasomes exert a distinct signal localization effect, attracting cells towards them. This phenomenon elucidates the migration and engulfment of migrasomes when HCCLM3-TSPAN4-GFP cells and Hep3B-TSPAN4-RFP cells are co-cultured.

To scrutinize the alterations in less invasive liver cancer cells subsequent to migrasome engulfment, we conducted a Transwell invasion experiment. Migrasomes from highly invasive HCCLM3 cells were extracted and added above the chamber, enabling less invasive Huh7 and Hep3B cells, alongside MHCC97H-CD151sh and HCCLM3-CD151sh cells, to engulf them. The results demonstrated a notable enhancement in the invasiveness of Huh7 and Hep3B cells, accompanied by a partial recovery of the invasive ability in MHCC97H-CD151sh and HCCLM3-CD151sh cells post-migrasome engulfment (Fig. 4d). Consequently, migrasomes function as localization signals, influencing and conditioning specific characteristics of liver cancer cells, particularly their invasiveness.

### CD151 promotes HCC migration through migrasome

**Fig. 5:**
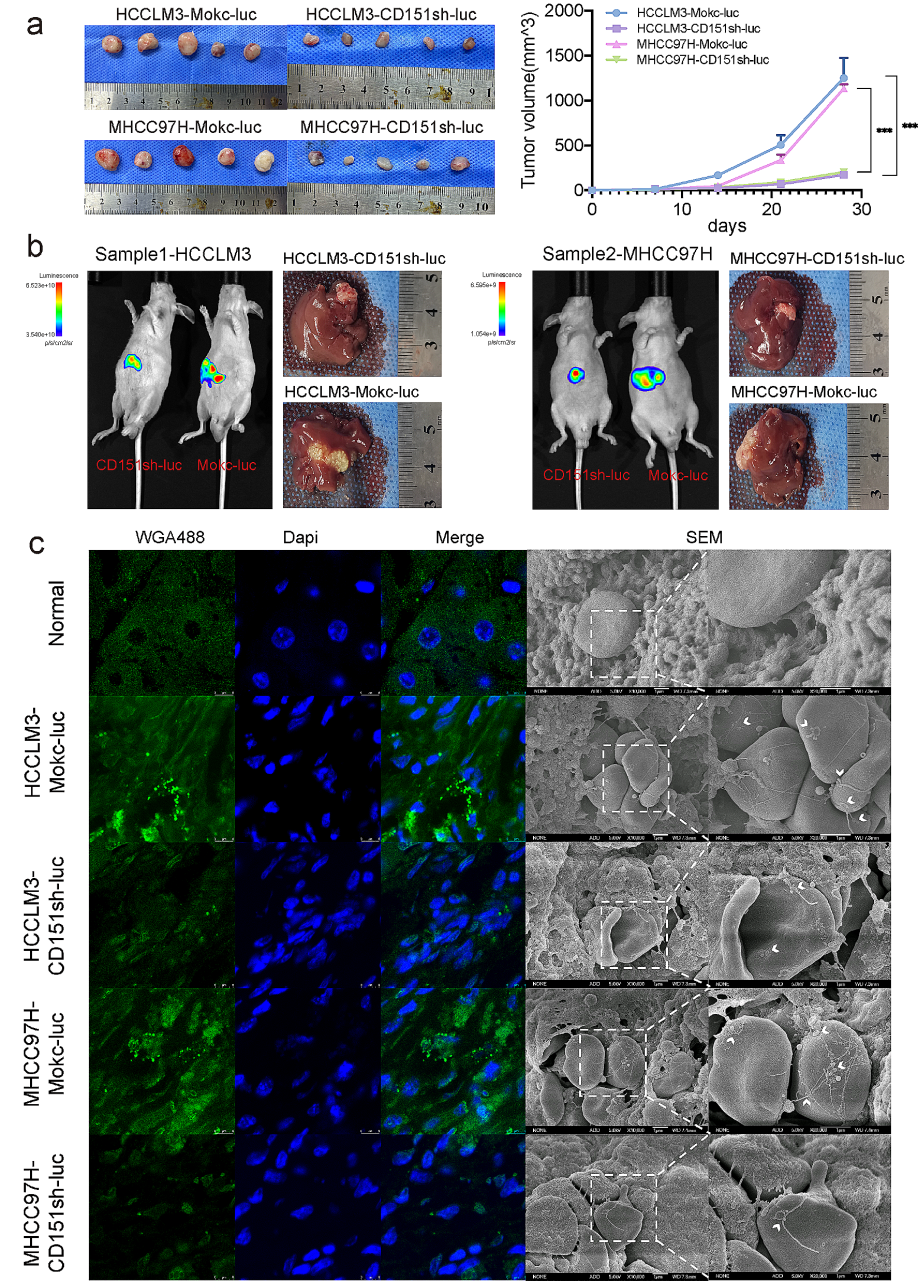
The influence of CD151 on migrasome generation and the development and metastasis of liver cancer in vivo. **a**: Cells treated with Mock shRNA or CDC151 shRNA were suspended in serum-free culture medium and injected into the upper abdomen of nude mice. Tumor volumes were measured weekly, and macroscopic observations of tumors were made after 28 days of transplantation. **b**: Subcutaneous tumors were cut into approximately 1.0 mm^3 pieces and orthotopically transplanted into the livers of nude mice. The mice were allowed to grow for three months, followed by intraperitoneal injection of D-Luciferin. This technique quantifies the number of tumor cells (total tumor burden), and color gradients indicate tumor sizes in vivo. Mice were then euthanized via cervical dislocation, and their livers were excised and photographed using a high-definition digital camera. **c**: Frozen sections of the above orthotopic tumors were stained with 2 µg/ml WGA-Alexa 488 and observed under confocal microscopy. SEM was then used to observe the processed orthotopic tumors. *p < 0.05; **p < 0.01; ***p < 0.001; ns, not significant

To assess the influence of CD151 on in vivo tumor growth, we employed two murine tumor models. Initially, 1 × 10^7 HCCLM3/MHCC97H-Mock-luc or HCCLM3/MHCC97H-CD151sh-luc cells were subcutaneously injected into BALB/c nude mice. After 4 weeks, D-Luciferin was intraperitoneally administered to quantify tumor cells (total tumor burden). Notably, the HCCLM3/MHCC97H-CD151sh-luc group exhibited a significant deceleration in in vivo tumor growth and reduced tumor volume compared to the HCCLM3/MHCC97H-Mock-luc group (Fig. 5a, S1a).

Subsequently, employing tumors generated as described above, we conducted an orthotopic tumor model experiment by implanting approximately 1 mm^3 tumor fragments into the livers of nude mice, allowing them to grow for 3 months before euthanasia through cervical dislocation. In line with the results from the subcutaneous tumor experiment, CD151 knockdown significantly impeded liver tumor growth and reduced tumor volume (Fig. 5b). Liver slices were stained, revealing a substantial presence of migrasome-like structures in the livers of HCCLM3/MHCC97H-Mock-luc group mice, while being scarce in the HCCLM3/MHCC97H-CD151sh-luc group (Fig. 5c). For a clearer observation of migrasomes in tissues, scanning electron microscopy (SEM) was employed to examine mouse livers from normal and orthotopic tumor tissues. The results exhibited a plethora of migrasome filamentous structures and migrasome-like structures in the HCCLM3/MHCC97H-Mock-luc group samples, while being less abundant in the HCCLM3/MHCC97H-CD151sh-luc group samples. Migrasome-like substances were challenging to capture in normal group samples (Fig. 5c).

Significantly, visible lung metastases were observed in mice from the HCCLM3-Mock and MHCC97H-Mock groups, while the lung tissues in the HCCLM3/MHCC97H-CD151sh group showed no apparent lung metastases (Figure S1b). Thus, high expression of CD151 can promote the generation of more migrasomes in vivo, thereby facilitating HCC cell migration.

### The migrasome affects angiogenesis

To unravel the biological processes orchestrated by CD151, contributing to the heightened metastasis and invasion traits in liver cancer cells, HCCLM3-CD151sh vs. HCCLM3-Mock and MHCC97H-CD151sh vs. MHCC97H-Mock cells underwent high-throughput sequencing analysis. Employing the R package edgeR [35] facilitated differential expression analysis, where genes meeting the criteria of FDR | log2(FoldChange) | > 1 and FDR < 0.05 were deemed significantly differentially expressed genes (Table S5-6).

**Fig. 6:**
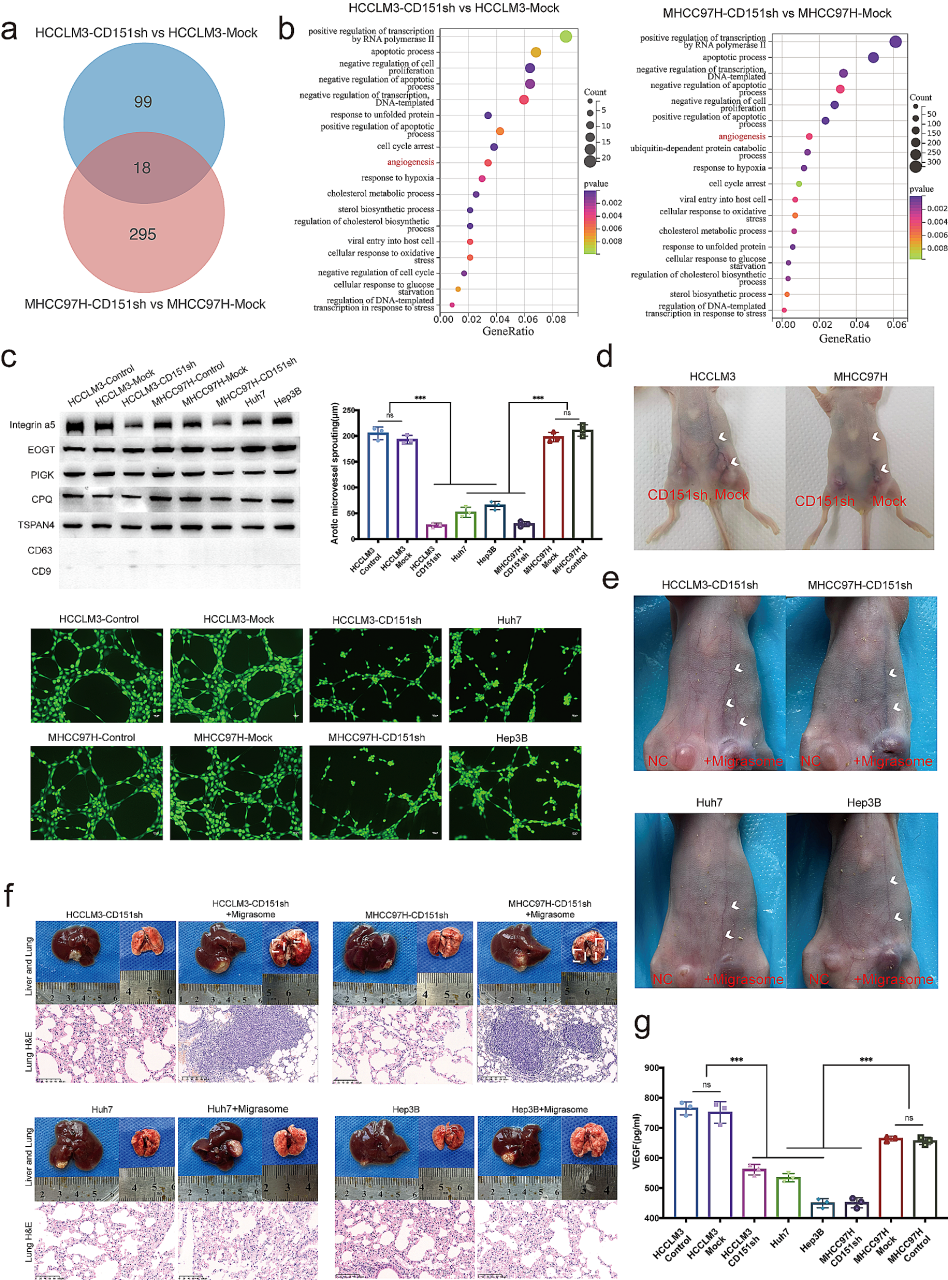
CD151 expression influences migrasome generation, thereby regulating tumor angiogenesis. **a**: Venn diagram depicting the common biological processes (BP) enriched by differentially expressed genes between HCCLM3-CD151sh vs. HCCLM3-Mock and MHCC97H-CD151sh vs. MHCC97H-Mock. **b**: Bubble plot illustrating the biological processes (BP) commonly enriched by differentially expressed genes between HCCLM3-CD151sh vs. HCCLM3-Mock and MHCC97H-CD151sh vs. MHCC97H-Mock, with bubble size indicating the count of genes and color depth representing the p-value magnitude. **c**: Western blot (WB) experiments validate markers in the extracted migrasomes. Matrigel assay determining the ability of HUVECs to form capillaries when cocultured with migrasomes collected from different liver cancer cell lines. **d**: Observation of neoangiogenesis induced by subcutaneous tumors injected with liver cancer cells into the inner thigh of mice. **e**: Cancer cells were co-injected with migrasomes into the subcutaneous region of the inner thigh in mice to observe the induction of angiogenesis. **f**: Mouse in situ tumor models were created using different cell lines, and migrasomes were injected into the mice to observe tumor growth and lung metastasis. H&E staining was used to display the pathological conditions of the mouse lungs. **g**: ELISA experiments to detect the content of VEGF in migrasomes extracted from different liver cancer cell lines. *p < 0.05; **p < 0.01; ***p < 0.001; ns, not significant

Subsequently, the differential genes of HCCLM3 and MHCC97H cells underwent Gene Ontology (GO) analysis, with significantly enriched results filtered based on a p-value < 0.01. The biological processes (BP) enriched by the two cell groups were intersected (Fig. 6a; Table S7-8), resulting in 18 common BPs (Table S9). Based on these 18 common BP pathways, a bubble plot was generated (Fig. 6b), revealing that only the “angiogenesis” pathway among the 18 BPs exhibited a certain correlation with liver cancer metastasis.

As a well-established contributor to early cancer metastasis, angiogenesis assumes a pivotal role. In the absence of angiogenesis, tumors typically attain a diameter of merely 1–2 mm, with cells beyond this range succumbing to nutritional deprivation [36]. Angiogenesis marks the shift from dormant to proliferative solid tumors, accelerating growth and promoting metastasis [37]. Consequently, the biological process of angiogenesis intricately links to the migration and invasion proclivity of liver cancer cells.

To validate these findings, we injected 1 × 10^7 HCCLM3/MHCC97H-Mock or HCCLM3/MHCC97H-CD151sh cells into the inner side of the hind limbs of mice. As subcutaneous tumors developed, liver cancer cells expressing CD151 normally (Mock group) exhibited substantial angiogenesis, characterized by thick and tortuous blood vessels. Conversely, the CD151 low-expression group (CD151sh group) induced angiogenesis, but the resulting blood vessels were slender and noticeably less prominent than those in the Mock group (Fig. 6d). Thus, CD151 evidently enhances angiogenesis in liver cancer.

Notably, research on migrasomes has elucidated that their promotion of angiogenesis stems from the substantial enrichment of VEGFA within migrasomes. Our hypothesis posits that CD151 influences migrasome generation, thereby impacting vascular remodeling and neovascularization in liver cancer. The validation of migrasomes’ role in neovascularization and vascular remodeling was undertaken through Matrigel analysis. After extracting migrasomes, we verified their associated markers [31] through Western blot experiments and ruled out the presence of exosomes using CD9 and CD63 as markers [33, 34]. Migrasomes extracted from distinct cells were introduced into incomplete culture medium, and HUVECs cultivation experiments revealed that the addition of migrasomes endowed the culture medium with the ability to induce the formation of capillaries by HUVECs (Fig. 6c). The vessel-like structures formed by HCCLM3-Control and MHCC97H-Control cell lines, characterized by heightened migrasome production, exhibited lengths of 199.12 ± 1.64 μm and 185.51 ± 5.30 μm, significantly surpassing those formed by Hep3B (62.16 ± 3.00 μm) and Huh7 (45.96 ± 2.51 μm) cell lines with fewer migrasomes and lower invasiveness.

Conversely, inhibiting the expression of CD151 in HCCLM3/MHCC97H-CD151sh cells led to a substantial decrease in migrasome production and resulted in vessel-like structures with reduced lengths (25.47 ± 0.84 μm/22.52 ± 1.75 μm). This impairment in neovascularization and vascular remodeling was evident when compared to HCCLM3/MHCC97H-Mock (187.81 ± 4.50 μm, 203.19 ± 5.90 μm) and HCCLM3/MHCC97H (199.12 ± 1.64 μm, 185.51 ± 5.30 μm) groups. To better observe the capabilities of migrasome in promoting angiogenesis and facilitating the metastasis of liver cancer in vivo, HCCLM3-CD151sh, MHCC97H-CD151sh, Huh7, and Hep3B cells were initially injected into the inner thighs of nude mice to form subcutaneous tumors. Subsequently, migrasomes extracted from highly invasive cells were injected near the tumors on one side every five days, while only PBS was injected on the control side. A noticeable extension of blood vessels was observed on the side where migrasomes were administered (Fig. 6e). Additionally, these cell lines were used to create orthotopic tumor models in mice, and migrasomes were injected into some mice every five days. Upon dissecting the mice, the tumors from HCCLM3-CD151sh (+ migrasome) and MHCC97H-CD151sh (+ migrasome) not only appeared larger but were also accompanied by pulmonary metastasis. While the tumors formed by Huh7 and Hep3B significantly increased in size due to the effect of migrasomes, no significant pulmonary metastasis occurred, potentially due to the lower intrinsic invasiveness of these two cell lines (Fig. 6f). Thus, migrasomes from highly invasive liver cancer cells can promote angiogenesis and distant metastasis in liver cancer. Previous research has indicated the richness of VEGF in migrasomes [18], suggesting their pivotal role in in vitro capillary formation and vascular remodeling. Substantiating this hypothesis, equal numbers of different cell lines were cultured for approximately 12–15 h, migrasomes were purified, resuspended in the same amount of incomplete culture medium, and ELISA experiments were conducted. The results (Fig. 6g) revealed the highest VEGF content in HCCLM3-Control, MHCC97H-Control, HCCLM3-Mock, and MHCC97H-Mock, with values of 765.22 ± 17.60 pg/ml, 656.48 ± 9.88 pg/ml, 751.32 ± 29.22 pg/ml, and 664.25 ± 6.51 pg/ml, respectively. Conversely, Hep3B, Huh7, HCCLM3-CD151sh, and MHCC97H-CD151sh exhibited markedly lower VEGF content, with values of 449.87 ± 12.65 pg/ml, 534.42 ± 11.31 pg/ml,561.23 ± 14.27 pg/ml, and 450.73 ± 13.63 pg/ml, respectively.

In summation, our study underscores that cell lines displaying heightened invasiveness exhibit elevated CD151 expression, produce more migrasomes, possess higher VEGF content, and demonstrate enhanced capabilities in promoting neovascularization and vascular remodeling. This provides compelling evidence for the pivotal role of CD151 in fostering neovascularization through migrasomes.

### CD151 and migrasome markers are associated with the prognosis and distant metastasis of HCC

**Fig. 7:**
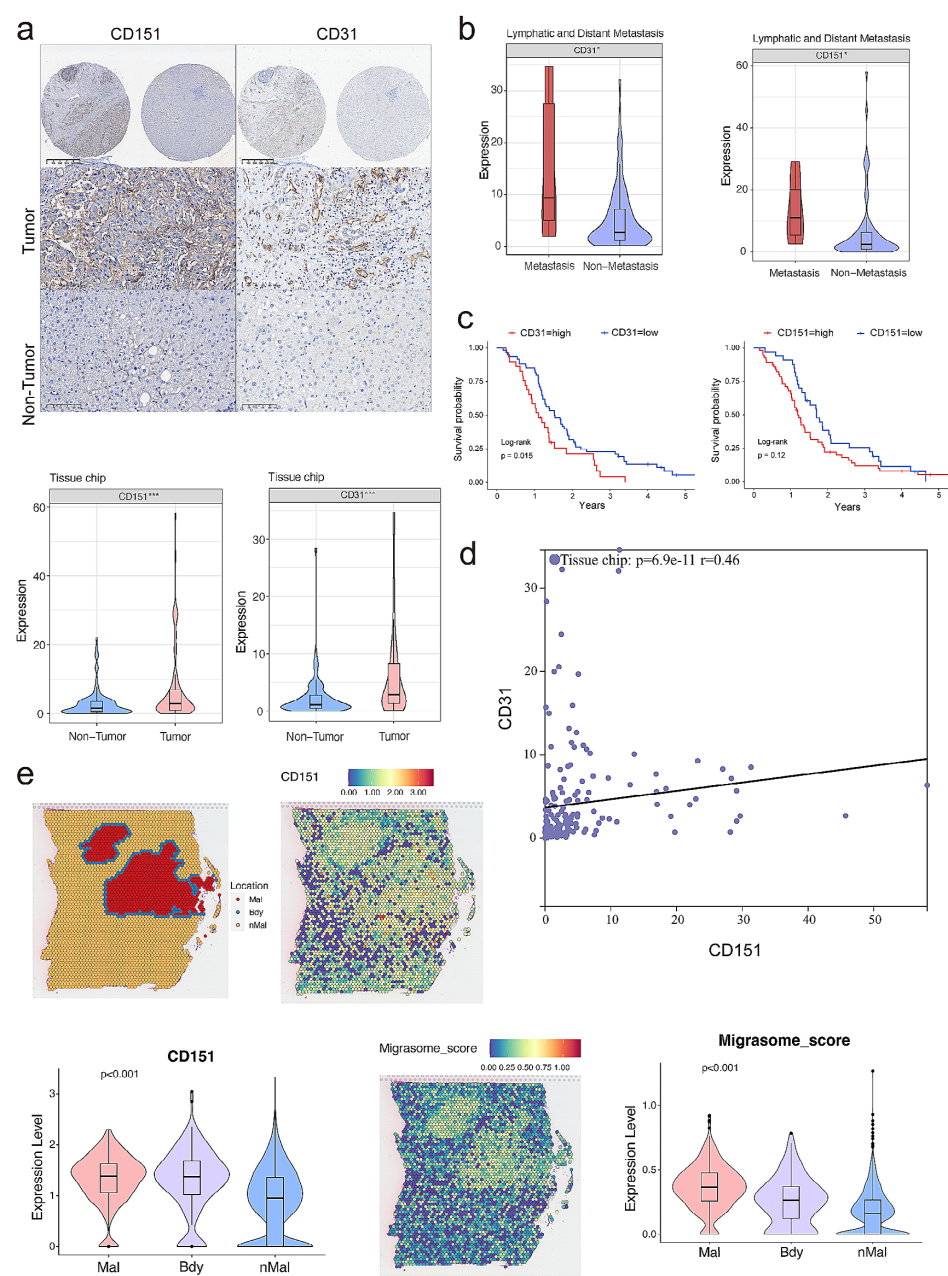
Correlation of CD151 with CD31 and migrasome Expression in HCC Tissues. **a**: Immunohistochemical correlation samples of CD151 and CD31 in HCC tissue microarrays (90 HCC tumor tissues and 90 adjacent non-tumor tissues). Immunohistochemical images of two representative samples are shown. The statistical graph represents the expression differences of CD151 and CD31 protein staining in tumor and non-tumor tissues on tissue microarrays. **b**: Statistical graph showing the expression differences of CD151 and CD31 in metastatic and non-metastatic tissues on tissue microarrays, where CD31 immunohistochemical staining represents angiogenesis in the samples. **c**: Survival curves of CD151 and CD31 in 90 clinical HCC samples. **d**: Correlation plot showing the expression correlation of CD151 and CD31 in samples on tissue microarrays. **e**: Expression localization of CD151 and migrasomes in liver cancer spatial transcriptome samples from the SpatialTME database, and their expression in Malignant, Boundary, and non-Malignant regions. *p < 0.05; **p < 0.01; ***p < 0.001; ns, not significant

Our investigation reveals a correlation between high CD151 expression and migrasome activity in liver cancer, indicating their involvement in angiogenesis and distant metastasis. To validate this association, we examined CD151 and CD31 expression in tumor tissues from 90 HCC patients using tissue microarray analysis. CD31, a marker for vascular endothelial cells, reflects angiogenesis levels. Our findings demonstrate significantly elevated protein expression levels of CD151 and CD31 genes in HCC tissues compared to adjacent normal tissues, indicating a higher CD151 expression in liver cancer tissues associated with increased vascularization (Fig. 7a).

Moreover, based on clinical data (Table S10), we categorized Lymphatic and Distant Metastasis as “Metastasis,” while the rest were deemed “Non-Metastasis.” Our analysis revealed higher CD151 expression in the “Metastasis” group, indicating a correlation between increased CD151 expression and distant metastasis (Fig. 7b). Additionally, CD31 expression was significantly higher in the “Metastasis” group compared to the “Non-Metastasis” group, suggesting greater angiogenesis in the former (Fig. 7b). Correlation analysis yielded a coefficient of *r* = 0.46 between CD31 expression and CD151, indicating a relationship between high CD151 expression and angiogenesis (Fig. 7d).

Furthermore, survival analysis unveiled a correlation between high CD31 expression and poor prognosis in liver cancer patients. However, due to sample size limitations, CD151 expression alone could not predict the survival of HCC patients (*P* > 0.05). Nonetheless, the overall trend suggests lower survival rates in HCC patients with high CD151 expression (Fig. 7c).

To comprehensively assess the correlation between migrasomes and CD151 expression, we examined the SpatialTME database (http://www.spatialtme.yelab.site) and found that CD151 is most expressed in the “Malignant” region of liver cancer spatial transcriptomic samples, followed by the “Boundary” region. Both regions exhibit higher expression values than the “Non-Malignant” region. Additionally, using Gene Set Variation Analysis (GSVA) to compute the expression of migrasome-related markers such as TSPAN4, NDST1, CPQ, EOGT, and TSPAN7, we observed an overlap between migrasome expression regions and CD151 expression, with both being highest in the “Malignant” and “Boundary” regions (Fig. 7e), indicating a strong correlation between CD151 and migrasome expression in liver cancer.

In summary, our findings suggest that CD151 expression mirrors migrasome localization and is significantly positively correlated with liver cancer angiogenesis and distant metastasis.

## Discussion

In exploring tumor cell dynamics, we identified migration bodies, vesicle-rich structures formed at the contracting tail of migrating cells, as key to understanding tumor metastasis. Our research highlights CD151 as a marker for these bodies, notably upregulated in hepatocellular carcinoma (HCC) compared to normal liver tissue. We demonstrate that CD151 is linked to migrasome formation, affecting the invasive capabilities of liver cancer cells. While previous work has linked migrasomes to various cellular functions including mitochondrial quality control [14, 15], substance transfer [32], signal integration [18], and angiogenesis [20], our findings provide new insights into their role in enhancing HCC’s metastatic potential.

Furthermore, our investigation posits migration bodies as signalling effectors. Migration bodies, generated by highly CD151-expressing HCC cell lines, exhibit a signal localisation effect on neighbouring liver cancer cells, attracting and facilitating their engulfment. Intriguingly, cells that engulf migration bodies acquire invasive capabilities akin to highly metastatic liver cancer cells. Hence, migration bodies play a role in conditioning early, non-highly invasive cells, eventually forming clusters of highly metastatic cells. These findings substantiate the significant contribution of migrasomes produced by CD151-expressing cells to the invasion and metastasis of liver cancer.

A prior research report identified the four-transmembrane protein TSPAN4 as a specific marker for migrasomes, impacting the generation of migration bodies. Although CD151, also a member of the four-transmembrane protein family, was not initially recognised as a marker for migration bodies by Huang et al. [38], our tumour-based study contradicts this, revealing significantly higher CD151 expression compared to TSPAN4. Experimental evidence substantiates CD151’s capability to label migration bodies, impacting their generation. Discrepancies in prior findings may arise from factors such as previous studies primarily conducted in NRK cells [39], with identified markers not universally validated across cell types, especially in tumour cells. The functional similarities shared by CD151 and TSPAN4, both belonging to the four-transmembrane protein family [40], elucidate CD151’s comparable labelling effect in tumour cells, where its expression significantly surpasses that of TSPAN4 in pan-cancer contexts.

This study highlights the influence of migrasome generation by CD151 on angiogenesis. CD151 activation triggers the release of pro-angiogenic factors such as nitric oxide via the PI3K/Akt pathway [41] and regulates integrin organization on endothelial cells, enhancing angiogenesis-related functions. Notably, CD151-null mice show reduced angiogenesis in pulmonary endothelial cells [42], suggesting CD151 as a target for anticancer therapy. Additionally, Zhang et al. emphasized migrasomes’ role in embryonic angiogenesis, especially those from monocytes containing VEGF [18]. While Shi et al. identified a connection between CD151 and neoangiogenesis in HCC, their findings on CD151’s effect on VEGF-dependent neoangiogenesis were inconclusive [43]. In contrast, Our results suggest that CD151 indirectly affects VEGF production, with migrasomes rich in VEGF playing a central role in angiogenesis, clarifying inconsistencies in prior research.

Our investigation posits the pivotal involvement of migrasomes in the vascular remodeling process within liver cancer particularly through the expression of CD151 and its correlation with migrasome activity and angiogenesis observed in our animal models. We identified a strong link between neovascularization and tumor progression, degeneration, infiltration, and metastasis. In the context of Hepatocellular Carcinoma (HCC), elevated CD151 levels correspond with increased migrasome marker (TSPAN4) levels, enhancing neovascular formation and promoting metastasis. Spatial metabolomic and tissue microarray analyses have further substantiated the association between high CD151 expression, increased neovascularization, and poor prognosis, providing critical insights into the mechanisms of tumor-induced neovascularization and its link to metastasis.

Although this study only delves into some functionalities of migrasomes in liver cancer, it offers valuable insights that can guide future research and clinical applications of migrasomes. For instance, migrasomes circulate in the bloodstream [44–46] and may appear in urine [47, 48], suggesting their utility in diagnosing cancer or identifying metastatic cell clusters. They also play a role in angiogenesis, indicating possibilities for targeting early tumor vascularization. Furthermore, similar to exosomes used in drug delivery [49–51], migrasomes might offer superior drug encapsulation and biocompatibility due to their larger size and cellular origin [52], potentially surpassing exosomes in effectiveness.

In summary, our study explores the complex relationship between CD151 and migrasome production, and the impact of migrasomes on angiogenesis in Hepatocellular Carcinoma (HCC) progression. Migrasomes facilitate more dynamic invasion and metastasis than individual cells, enabling enhanced intercellular communication and manipulation of cellular functions via blood or bodily fluids, which intensifies tumor progression. These findings highlight migrasomes as critical to the invasive and metastatic capabilities of HCC and as potential targets for anti-HCC therapy.

### Electronic supplementary material

Below is the link to the electronic supplementary material.

Supplementary Material 1

Supplementary Material 2

Supplementary Material 3

## Data Availability

Data will be made available on request. The raw data of cell sequencing has been uploaded to figshare database (DOI: 10.6084/m9.figshare.25182404). All the data sets used in this study including TCGA dataset, ICGC dataset, GEO dataset and DepMap dataset are publicly available.

## Abbreviations

TSPAN4: Tetraspanin 4
HCC: Hepatocellular carcinoma
mRNA: Messenger RNA
OS: Overall survival
qRT-PCR: Quantitative real-time polymerase chain reaction
VEGF: Vascular Endothelial Growth Factor
LIHC: Liver Hepatocellular Carcinoma
TCGA: The Cancer Genome Atlas
ICGC: International Cancer Genome Consortium
ATCC: American Type Culture Collection
GEO: Gene Expression Omnibus
HUVECs: Human umbilical vein endothelial cells
SD: Standard deviation
TEMs: Tetraspanin Enriched Microdomains
EMT: Epithelial-mesenchymal transition
SEM: Scanning electron microscopy
GO: Gene Ontology
BP: Biological processes
