# Supplementary material for: CD151-enriched migrasomes mediate hepatocellular carcinoma invasion by conditioning cancer cells and promoting angiogenesis
Source: J Exp Clin Cancer Res. 2024 Jun 6;43:160. doi: 10.1186/s13046-024-03082-z (PMC11155183; doi:10.1186/s13046-024-03082-z)
Supplement: Supplementary file 1 — Supplementary Material 1 [file 13046_2024_3082_MOESM1_ESM.docx]

**
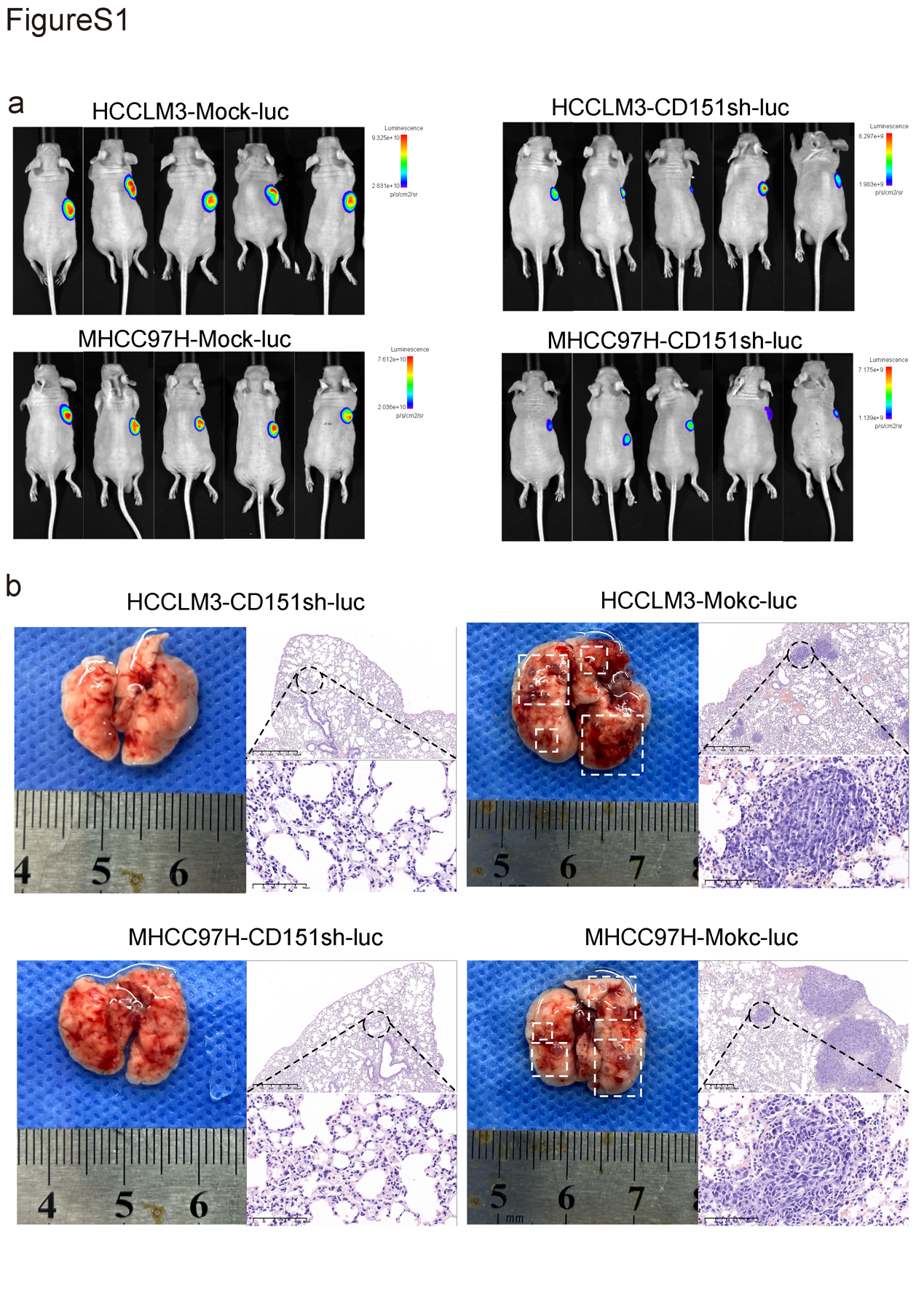
Figure S1:**

Subcutaneous tumors and lung metastasis in experimental mice.

a: Cells treated with Mock shRNA or CDC151 shRNA were suspended in serum-free culture medium and injected into the upper abdomen of nude mice. D-Luciferin is intraperitoneally injected. This technology allows quantification of the amount of tumor cells (total tumor burden), which allows development of tumor to be assessed ex vivo. The color gradient represents the size of the tumor in vivo. b: The lung metastasis rate of the HCCLM3/MHCC97H-Mock-luc group was 100% per mouse, while the HCCLM3/MHCC97H-CD151-luc group had almost no lung metastasis. Mouse lungs, both metastatic and non-metastatic, were photographed using a high-definition digital camera, and H&E staining was used to identify lung metastases.
